# Drosophila DNA polymerase theta utilizes both helicase-like and polymerase domains during microhomology-mediated end joining and interstrand crosslink repair

**DOI:** 10.1371/journal.pgen.1006813

**Published:** 2017-05-25

**Authors:** Kelly Beagan, Robin L. Armstrong, Alice Witsell, Upasana Roy, Nikolai Renedo, Amy E. Baker, Orlando D. Schärer, Mitch McVey

**Affiliations:** 1 Department of Biology, Tufts University, Medford, Massachusetts; 2 Department of Chemistry and Department of Pharmacological Sciences, Stony Brook University, Stony Brook, New York, United States of America; 3 Center for Genomic Integrity, Institute for Basic Science, Ulsan, Korea and Department of Biological Sciences, School of Life Sciences, Ulsan National Institute of Science and Technology, Ulsan, Korea; Columbia University, UNITED STATES

## Abstract

Double strand breaks (DSBs) and interstrand crosslinks (ICLs) are toxic DNA lesions that can be repaired through multiple pathways, some of which involve shared proteins. One of these proteins, DNA Polymerase θ (Pol θ), coordinates a mutagenic DSB repair pathway named microhomology-mediated end joining (MMEJ) and is also a critical component for bypass or repair of ICLs in several organisms. Pol θ contains both polymerase and helicase-like domains that are tethered by an unstructured central region. While the role of the polymerase domain in promoting MMEJ has been studied extensively both *in vitro* and *in vivo*, a function for the helicase-like domain, which possesses DNA-dependent ATPase activity, remains unclear. Here, we utilize genetic and biochemical analyses to examine the roles of the helicase-like and polymerase domains of Drosophila Pol θ. We demonstrate an absolute requirement for both polymerase and ATPase activities during ICL repair *in vivo*. However, similar to mammalian systems, polymerase activity, but not ATPase activity, is required for ionizing radiation-induced DSB repair. Using a site-specific break repair assay, we show that overall end-joining efficiency is not affected in ATPase-dead mutants, but there is a significant decrease in templated insertion events. *In vitro*, Pol θ can efficiently bypass a model unhooked nitrogen mustard crosslink and promote DNA synthesis following microhomology annealing, although ATPase activity is not required for these functions. Together, our data illustrate the functional importance of the helicase-like domain of Pol θ and suggest that its tethering to the polymerase domain is important for its multiple functions in DNA repair and damage tolerance.

## Author summary

Error-prone DNA Polymerase θ (Pol θ) plays a conserved role in a mutagenic DNA double-strand break repair mechanism called microhomology-mediated end joining (MMEJ). In many organisms, it also participates in a process crucial to the removal/repair of DNA interstrand crosslinks. The exact mechanism by which Pol θ promotes these processes is unclear, but a clue may lie in its dual-domain structure. While the role of its polymerase domain has been well-studied, the function of its helicase-like domain remains an open question. Here we report an absolute requirement for ATPase activity of the helicase-like domain during interstrand crosslink repair in *Drosophila melanogaster*. We also find that although end joining frequency does not decrease in ATPase-dead mutants, ATPase activity is critical for generating templated insertions. Using purified Pol θ protein, we show that it can bypass synthetic substrates mimicking interstrand crosslink intermediates and can promote MMEJ-like reactions with partial double-stranded and single-stranded DNA. Together, these data demonstrate a novel function for the helicase-like domain of Pol θ in both interstrand crosslink repair and MMEJ and provide insight into why the dual-domain structure has been conserved throughout evolution.

## Introduction

DNA double strand breaks (DSBs) and interstrand crosslinks (ICLs) compromise both strands of the DNA duplex and must be repaired to ensure cellular survival. While DSBs disrupt the physical integrity of DNA, ICLs tether DNA strands together through a covalent bond. Thus, both types of lesions can impede critical processes such as replication and transcription. ICLs require a complex network of proteins for their removal and involve multiple DNA repair pathways (reviewed in [1, 2]). Because repair of ICLs can generate one- or two-ended DSB intermediates, the identification of proteins that are involved in both ICL and DSB repair might provide insight into their common mechanisms [3, 4].

In *Drosophila melanogaster*, DNA Polymerase θ (Pol θ) has emerged as one of these dual-function proteins. It was first identified in a mutagen sensitivity screen where mutations in *mus308*, the gene encoding Pol θ, caused hypersensitivity to ICL-inducing agents but not to other DNA alkylating agents [5, 6]. Pol θ is an A-family polymerase with homology to *E*. *coli* Pol I and contains an N-terminal helicase-like domain connected to the polymerase domain through a long, unstructured central domain [7–9]. Both the polymerase and helicase domains are conserved among metazoans. In vertebrates, the polymerase domain also contains three insertion loops that are not present in Pol I and which are poorly conserved in invertebrates (reviewed in [10]). Early work on *Drosophila* Pol θ showed that it likely acts downstream of ICL “unhooking” [5], where one strand of the DNA helix is incised on both sides of the crosslink. Subsequent studies in *C*. *elegans* revealed a conserved role for Pol θ in ICL repair [11]. However, the mechanisms by which Pol θ promotes ICL repair remain uncharacterized.

Numerous studies have shown that Pol θ is also critical for alternative end-joining repair of DSBs via a pathway named microhomology-mediated end joining (MMEJ) [12–17]. MMEJ occurs when short, complementary DNA sequences located at broken DNA ends anneal and serve as primers for fill-in synthesis. An early study of Pol θ-mediated MMEJ in Drosophila, using a transposon-induced gap repair system, demonstrated that repair involving annealing of 5–10 nucleotide pre-existing microhomologies was dependent on Pol θ [18]. Pol θ has also been shown to promote MMEJ in *C*. *elegans* [19, 20], zebrafish [21], and mice [13].

While MMEJ acts independently of classical NHEJ proteins, 5’→3’ resection stimulates MMEJ and there is some overlap between proteins involved in the intial steps of both MMEJ and homologous recombination [22]. Following resection, microhomologies located internal to the break site can also be used for annealing, although this requires trimming of non-homologous flaps prior to synthesis [23]. *In vitro*, the polymerase domain of Pol θ is sufficient to join DNA ends with limited microhomologies, though the optimal length of DNA overhangs varies by system [12, 13]. Additionally, the polymerase domain can mediate the alignment of both internal and terminal microhomologies and displace annealed DNA during template extension *in vitro* [12].

In addition to the use of microhomologies, another signature of many alternative end-joining events in metazoans is the presence of small insertions that appear to be derived from flanking DNA sequences [24]. It is now clear that Pol θ is often utilized during the generation of these templated insertions. In Pol θ-null mouse B cells, programmed DSBs formed during class-switch recombination are repaired at a similar rate to wildtype but lack insertions of >1 nt, indicating that Pol θ is required to generate these insertions [13]. Studies with I-*Sce*I induced chromosomal DSBs in Drosophila documented repair events with >4 nt templated insertions that were largely dependent on Pol θ [25]. In addition, Pol θ plays a critical role in the generation of insertions during alternative end joining repair of collapsed replication forks in *C*. *elegans* [19, 20, 26] and during T-DNA insertion in Arabidopsis [27].

Though the function of the polymerase domain of Pol θ has been studied extensively *in vitro*, thus far no defined role exists for the helicase-like domain. The helicase-like domain displays DNA-dependent ATPase activity *in vitro*, but DNA unwinding activity has not been demonstrated *in vitro* or *in vivo* [8, 28]. Pol θ null mouse embryonic fibroblasts are extremely sensitive to the DSB-inducing agent bleomycin and the addition of the polymerase domain alone is sufficient to restore resistance to these cells [13]. The helicase-like domain, therefore, does not appear to be required for efficient DSB repair in mammalian cells and its vital role(s), if any, in DNA metabolism have remained largely uncharacterized.

Here, we report our efforts to further elucidate the roles of the helicase-like and polymerase domains of Drosophila Pol θ in ICL and DSB repair. We show that purified Pol θ can bypass model unhooked ICL substrates and promote the initial steps of MMEJ *in vitro*, with minimal requirement for ATPase activity. In contrast, our *in vivo* experiments establish that both the ATPase activity of the helicase-like domain and DNA polymerase activity are critical for tolerance or repair of ICLs and for the generation of templated insertions during alternative end joining. Tethering of these two enzymatic domains may therefore promote complex DNA transactions necessary for both types of repair.

## Results

### Pol θ ATPase activity is required for resistance to a DNA interstrand crosslinking agent but not ionizing radiation

**Fig 1:**
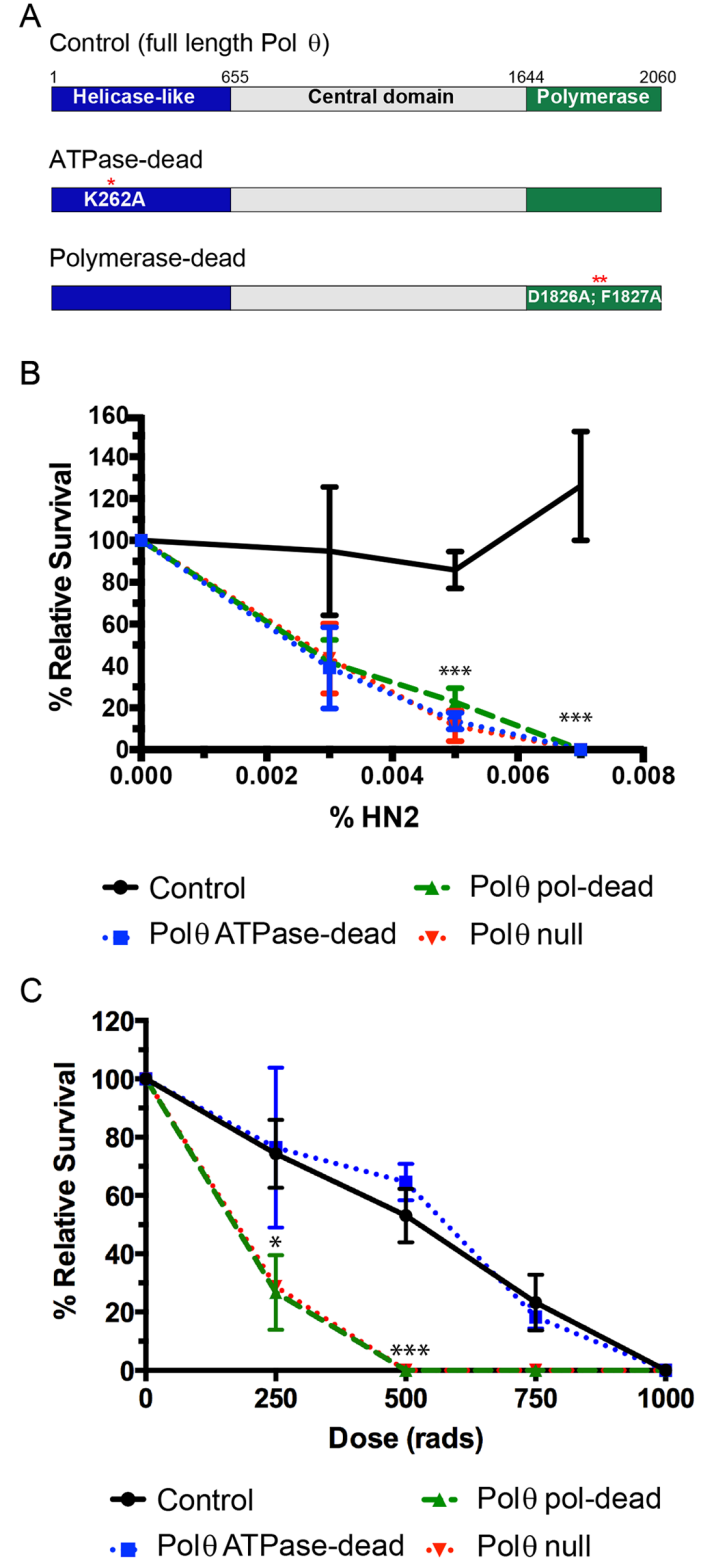
*Drosophila* Pol θ ATPase activity is required for resistance to a DNA interstrand crosslinking agent but not ionizing radiation. **A**. Schematic of *mus308* alleles and transgenes. The N-terminal helicase-like domain (blue) and C-terminal polymerase domain (green) are connected by the central domain (gray). Control, ATPase-dead, and polymerase-dead (pol-dead) are transgenic alleles. Numbers indicate amino acid positions in the protein. **B**. Nitrogen mustard sensitivity survival curve for domain-specific mutants. Transgenes were present in a *mus308Δ* background. **C**. Ionizing radiation sensitivity survival curve for domain-specific transgenic mutants. All transgenes were in a *mus308Δ*, *spn-A* (*rad51*) background. Data points represent the average of three independent trials and standard deviations are shown. *p<0.05, **p<0.01, ***p<0.0001 compared to full-length control, two-way ANOVA, Tukey’s post-hoc test.

Drosophila with mutations in the *mus308* gene, encoding Pol θ, were originally identified by their hypersensitivity to the bi-functional alkylating agent nitrogen mustard, which induces ICLs [5]. To determine which enzymatic functions of Pol θ are required during ICL repair, we generated a series of transgenic Drosophila lacking the endogenous *mus308* gene and possessing Pol θ transgenes with a mutation in the Walker A box of the helicase-like domain that prevents ATP binding (K262A; ATPase-dead) or the catalytic function of the polymerase domain (D1826A, F1827A; pol-dead) (Fig 1A). In addition, we created a transgenic rescue stock with a full-length wildtype copy of Pol θ. All of the transgenes included the full-length *mus308* promoter to ensure endogenous expression.

We exposed larvae from these transgenic stocks to increasing concentrations of nitrogen mustard and quantified survival to adulthood. Similar to previous studies, we found that homozygous mutants lacking Pol θ are hypersensitive to nitrogen mustard, as compared to heterozygous controls (Fig 1B). Addition of the full-length transgene rescued this sensitivity to wild-type levels. Pol-dead mutants were hypersensitive to low concentrations of nitrogen mustard, indicating a requirement for DNA synthesis in Pol θ-mediated ICL repair. Intriguingly, we found that ATPase-dead mutants were equally as sensitive to nitrogen mustard as pol-dead mutants or flies lacking Pol θ.

In mammals, the polymerase activity of Pol θ is required for double-strand break repair, specifically for MMEJ [12, 13, 17]. Interestingly, the polymerase domain of purified Pol θ protein alone is sufficient to join DNA ends *in vitro*, indicating that ATPase activity is dispensable [12]. To test whether this is also true in Drosophila, we exposed flies expressing different Pol θ transgenes to ionizing radiation (IR) in the absence of endogenous Pol θ. Our previous study showed that flies lacking Pol θ are only sensitive to IR in the absence of homologous recombination [18]. Therefore, we conducted these experiments in flies lacking RAD51 (encoded by the *spn-A* gene). As expected, *mus308Δ*, *spn-A* mutants were highly sensitive to IR and addition of the full-length transgene rescued this sensitivity (Fig 1C). Indeed, a dose of 500 rads was lethal to mutants lacking Pol θ and RAD51, while 50 percent of *spn-A* mutants were able to survive this exposure. Pol-dead and Pol θ null mutants were equally hypersensitive to IR. However, ATPase-dead mutants behaved identically to the wild-type control. Thus, the ATPase activity of Pol θ is needed for survival following exposure to DNA interstrand crosslinking agents, but not for damage induced by ionizing radiation.

### Pol θ can bypass unhooked ICL intermediates *in vitro*

To further delineate the functions of the ATPase and polymerase domains in ICL repair, we turned to an *in vitro* system. We purified full-length wild-type, ATPase-dead, and pol-dead proteins to near homogeneity from insect cells. SDS-PAGE and Western blotting confirmed the identity of full-length Pol θ, although some minor degradation products were visible (S1 Fig).

**Fig 2:**
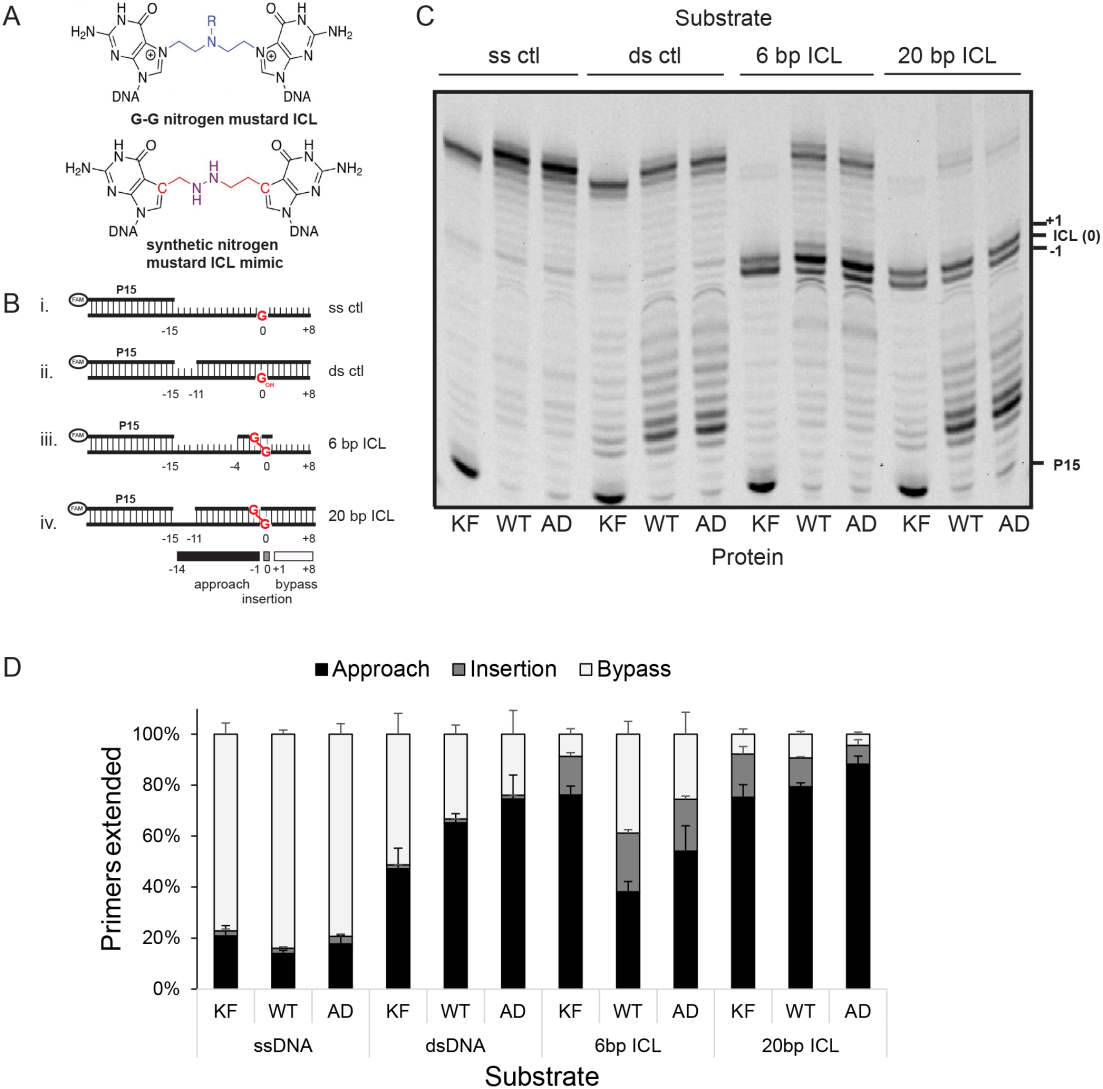
Efficient bypass of interstrand crosslinks by Pol θ. **A**. Structures of a nitrogen mustard interstrand crosslink (ICL) between two guanines and a 5-atom synthetic nitrogen mustard ICL mimic. **B**. Substrates used in the primer extension assays. 6-FAM labeled primer was annealed to different templates: (i.) single-stranded control containing ICL precursor G, (ii.) double-stranded control with ICL precursor G, (iii.) ICL substrate in a 6 bp duplex, (iv.) ICL substrate in a 20 bp duplex. Red highlighting indicates ICL precursor G (G_OH_) or crosslinks. **C**. Comparison of ICL bypass between Klenow fragment (KF), wild-type Pol θ (WT), and ATPase-dead Pol θ (AD). 5 nM of control or ICL templates were incubated with 1 nM Klenow for 5 min or 0.2 nM Pol θ for 10 min. at 37°C. Products were separated by denaturing PAGE on a 10% gel. **D**. Quantification of bypass. Each lane was divided into approach (-14 to -1), insertion (0) and bypass (+1 to +8) segments and corresponding band intensities expressed as a percentage of all the products combined. Data represent the mean of three experiments and error bars indicate standard deviations.

Current models suggest that during replication-coupled ICL repair, two incisions are made on either side of the ICL to release it from one strand, generating an “unhooked” ICL in which the excised fragment remains attached to other strand through the ICL itself. However, the exact location of the incisions and the length of the remaining olionucleotide attached to the duplex are currently unknown [29]. Thus, *in vitro* studies of ICL bypass typically use model unhooked ICLs of different lengths [30]. We used our purified Pol θ proteins in primer extension assays with substrates mimicking 6 base pair (bp) or 20 bp unhooked nitrogen mustard ICLs and corresponding control substrates (Fig 2A and 2B) [31, 32]. Both wild-type and ATPase-dead Pol θ were able to effectively extend a fluorescently labeled primer on a single-strand control template, although their activity on a partial double-strand control template was somewhat reduced compared to the Klenow fragment of *E*. *coli* DNA polymerase I (Fig 2C and 2D). While both Klenow fragment and wild-type Pol θ could insert a nucleotide opposite an ICL, Klenow was unable to extend past the ICL with both the 6 bp and 20 bp unhooked crosslinks (Fig 2C and 2D). By contrast, Pol θ was able to efficiently carry out extension to full product for the 6 bp ICL. The efficiency of extension was reduced with the 20 bp ICL, similar to previous reports with other polymerases [31, 32]. We observed only moderately reduced extension activity with ATPase-dead Pol θ compared to wild-type, suggesting that ATP hydrolysis is not required for bypassing an unhooked ICL *in vitro*.

### Alternative end joining is reduced in the absence of Pol θ polymerase activity

**Fig 3:**
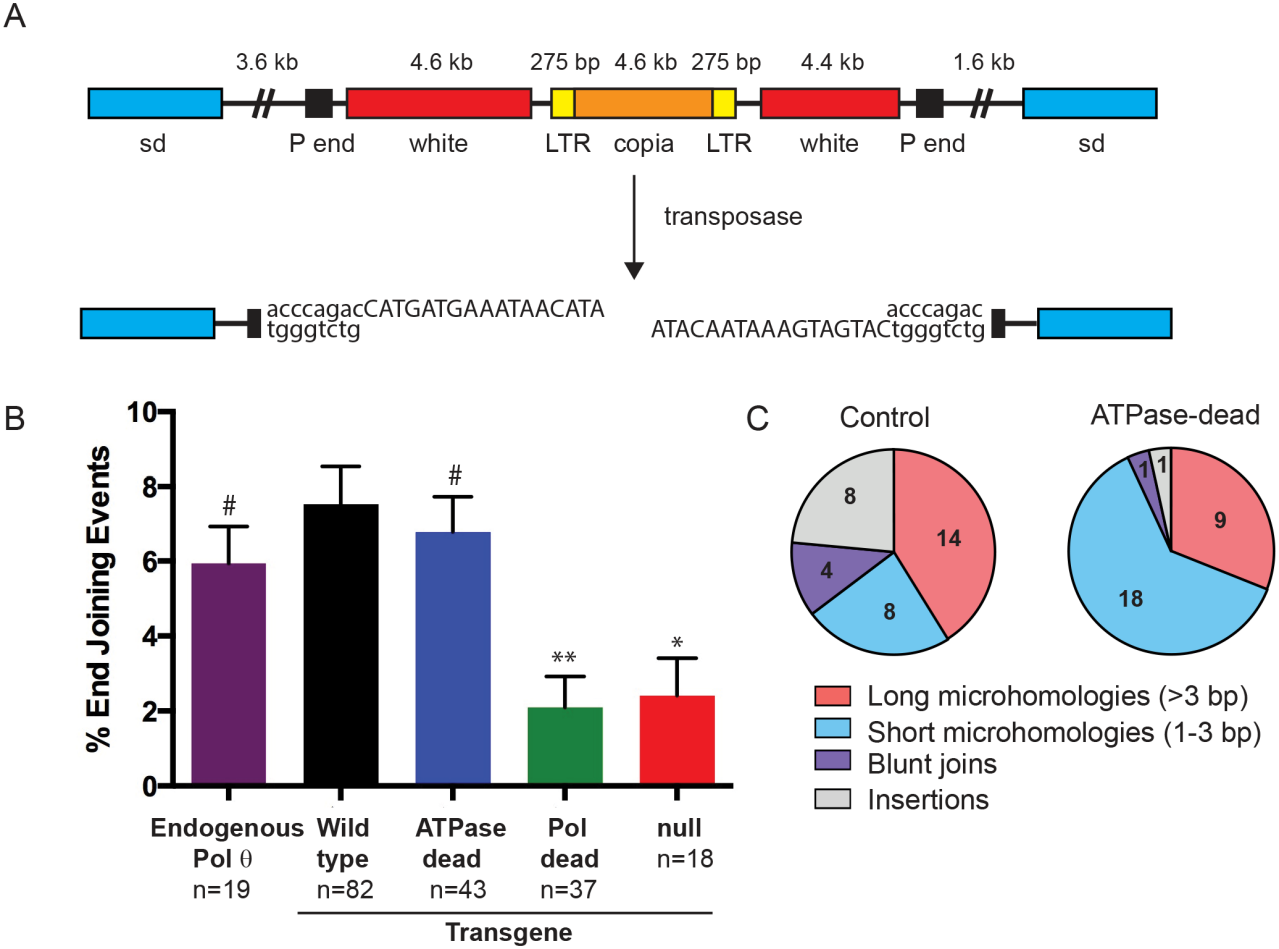
Pol θ ATPase activity does not affect end joining frequency but promotes the formation of complex insertions. **A**. Schematic of the *P{w*^*a*^*}* construct (top). After expression of transposase, the P-element is excised leaving 17 nt overhangs (bottom). **B**. Frequency of end joining repair in domain-specific transgenic alleles and controls. All transgenes are in a *mus308Δ*, *spn-A* (*rad51*) background. The number of independent vials (n) represented by each bar and the standard error of the mean is shown. **C**. Summary of junction types recovered in control and ATPase-dead transgenic alleles. *p<0.05, **p<0.01, # p = n.s. compared to wild-type control, two-way ANOVA, Tukey’s post hoc test.

Previous work from our lab showed that hypomorphic and point mutations in Pol θ lead to a significant decrease in DNA ligase 4-independent alternative end joining [18]. During Pol θ-mediated end joining of I-*Sce*I or *P* element-induced double-strand breaks, microhomologies are often used to align DNA ends and insertions are present in approximately 25% of repair junctions [18, 25]. To determine which domains of Pol θ might be involved in the generation of these insertions, we utilized a well-characterized site-specific gap repair assay [33]. In this assay, a 14 kilobase transposon (*P{w*^*a*^*}*) is inserted into an intron of the *scalloped* gene on the *X* chromosome. Excision of *P{w*^*a*^*}* is catalyzed by *P* transposase, resulting in a two-ended double-strand break with 17-nucleotide non-complementary 3’ overhangs (Fig 3A). While this break can be repaired proficiently through homologous recombination, in the absence of RAD51 the break is repaired solely through end joining. Repair events that occur in the pre-meiotic male germline can be recovered from the female progeny that inherit the repaired chromosome and sequenced.

As expected, expression of wild-type Pol θ in a *mus308Δ* mutant background resulted in a similar frequency of alternative end-joining events to flies expressing endogenous Pol θ (Fig 3B). Ablation of ATPase activity did not affect the frequency of end joining repair (Fig 3B), in agreement with previously published results that ATPase function is not required for MMEJ [12, 13]. In contrast, we saw a significant decrease in end joining with the pol-dead allele, comparable to *mus308Δ* mutants.

In the *P{w*^*a*^*}* system, repair events accompanied by large deletions (>1.5 kilobases) that remove part of the coding region of the *scalloped* gene can be scored by the presence of female progeny possessing scalloped wings [34]. These deletions are thought to occur through a resection-based mechanism and were previously observed in flies with Pol θ-inactivating point mutations [18]. Notably, we observed a substantial increase in deletion-associated repair events in the polymerase-dead mutant compared to the full-length control and ATPase-dead mutant (S1 Table). Thus, our data suggest that Pol θ polymerase, but not ATPase activity, is required to promote normal levels of alternative end joining and to suppress alternative, deletion-prone repair pathways.

### Complex insertions generated during alternative end joining depend upon both ATPase and polymerase activities of Pol θ

Analysis of the repair junctions recovered from the *P{w*^*a*^*}* assay showed that Pol θ-proficient flies often utilized long microhomologies during break repair. We saw a particularly high usage of an 8 bp imperfect internal microhomology in both the wildtype and the ATPase-dead backgrounds (Tables 1 and 2). In addition, we recovered many repair products from both backgrounds that appeared to be generated through annealing at short microhomologies, with a preference for microhomologies closer to the 3’ ends of the single-stranded overhangs. Thus, it appears that ATPase activity is not required to generate products through annealing at pre-existing microhomologies.

**Table 1:** Control *P{w*^*a*^*}* repair junctions.

Type of repair event	Sequence 5’ of break	Microhomology/ insertion	Sequence 3’ of break	# of isolates
**Original sequence**	acccagac*CATGATGAAATAACATA*	-	*TATGTTATTTCATCATG*acccagac	-
**Long microhomology (>3 bp)**				
	acccagac	(CATgATGA)	cccagac	10
	acccagac	(CATGA)	cccagac	4
**Short microhomology (1–3 bp)**				
	acccagac*CATG*	(ATG)	*TTATTTCATCATG*acccagac	1
	acccagac*CATGATGAAATAA*	(CAT)	*CATG*acccagac	1
	acccagac*CATGATGAAATAAC*	(AT)	*GTTATTTCATCATG*acccagac	3
	acccagac*CATGATGAAATAACA*	(TA)	*TGTTATTTCATCATG*acccagac	1
	acccagac*CATGATG*	(A)	*TG*acccagac	1
	acccagac*CATGATGAAATAACAT*	(A)	*TGTTATTTCATCATG*acccagac	1
**Blunt join**				
	acccagac*CATGATGAAATAACATA*	-	*ATGTTATTTCATCATG*acccagac	1
	acccagac*CATGATGAAATAACATA*	-	*TATGTTATTTCATCATG*acccagac	3
**Insertion**				
	acccagac*CATGATGAAATAACATA*	TGTTATATGT	*TATGTTATTTCATCATG*acccagac	1
	acccagac*CATGATGAAATAACAT*	GTTATGTTATT	*TATGTTATTTCATCATG*acccagac	1
	acccagac*CATGATGAA*	GCCTC	acccagac	1
	acccagac*CATGATGAAATAACA*	TGTTA	*TGTTATTTCATCATG*acccagac	1
	acccagac*CATGATGAAATAACA*	ACA	*TATGTTATTTCATCATG*acccagac	1
	acccagac*CATGATGAAATAACATA*	ACA	*TGTTATTTCATCATG*acccagac	1
	acccagac*CATGATGAAATAACAT*	GTA	*TGTTATTTCATCATG*acccagac	1
	acccagac*CATGA*	CC	acccagac	1

Sequences of repair events showing microhomology usage and insertions obtained from Pol θ-proficient flies. Microhomologies are indicated by parentheses, with imperfect microhomologies shown in lower case. Sequences from endogenous Pol θ and full-length controls are pooled.

**Table 2:** ATPase-dead *P{w*^*a*^*}* repair junctions.

Type of repair event	Sequence 5’ of break	Microhomology/ insert	Sequence 3’ of break	# of isolates
**Original sequence**	acccagac*CATGATGAAATAACATA*	-	*TATGTTATTTCATCAT*Gacccagac	-
**Long microhomology (>3 bp)**				
	acccagac	(CATgATGA)	cccagac	5
	acccagac	(CATcATGA)	cccagac	2
	acccagac*C*	(ATGaT)	*ATTTCATCATG*acccagac	1
	acccagac*CATG*	(ATGA)	cccagac	1
**Short microhomology (1–3 bp)**				
	acccagac*CATGATGAAATAA*	(CAT)	*CATG*acccagac	3
	acccagac*CATGATGAAATAA* acccagac*C*	(CAT) (ATG)	*G*acccagac *TTATTTCATCAT*Gacccagac	1 1
	acccagac*CATGATGAAATAAC*	(AT)	*GTTATTTCATCATG*acccagac	9
	acccagac*CATGATGAAA*	(TA)	*TTTCATCATG*acccagac	2
	acccagac*CATGATG*	(A)	*TG*acccagac	1
	acccagac*CATGATGAAATAACA*	(T)	*TATTTCATCATG*acccagac	1
**Blunt join**				
	acccagac*CATGATGAAATAACATA*	-	*TTATTTCATCATG*acccagac	1
**Insertion**				
	acccagac*CATGATGAAATAACAT*	GTTA	*GTTATTTCATCATG*acccagac	1

Sequences of repair events showing microhomology usage and insertions obtained from ATPase-dead mutants. Microhomologies are indicated by parentheses, with imperfect microhomologies shown in lower case.

We also observed frequent insertions in wild-type repair products that appeared to be templated from sequences near the break site. This insertion class accounted for just 3% of repair junctions recovered from the ATPase-dead mutants, significantly less than the 24% of wild-type repair junctions with insertions (p = 0.03, Fisher’s exact test; Tables 1 and 2 and Fig 3C). The sole insertion event generated in the ATPase-dead mutant was a four-nucleotide insertion apparently templated from sequence immediately adjacent to the break. In contrast, repair events in flies with endogenous *mus308* expression or the control transgene contained insertions templated from sequences immediately adjacent to the break as well as internal sequences (Table 1). Flies expressing wild-type Pol θ also contained more complex events that can be explained by an iterative process of multiple rounds of synthesis, dissociation, and reannealing. These events were absent in ATPase-dead mutants.

### Pol θ ATPase and polymerase activities are both used during annealing and extension of ssDNA molecules *in vitro*

**Fig 4:**
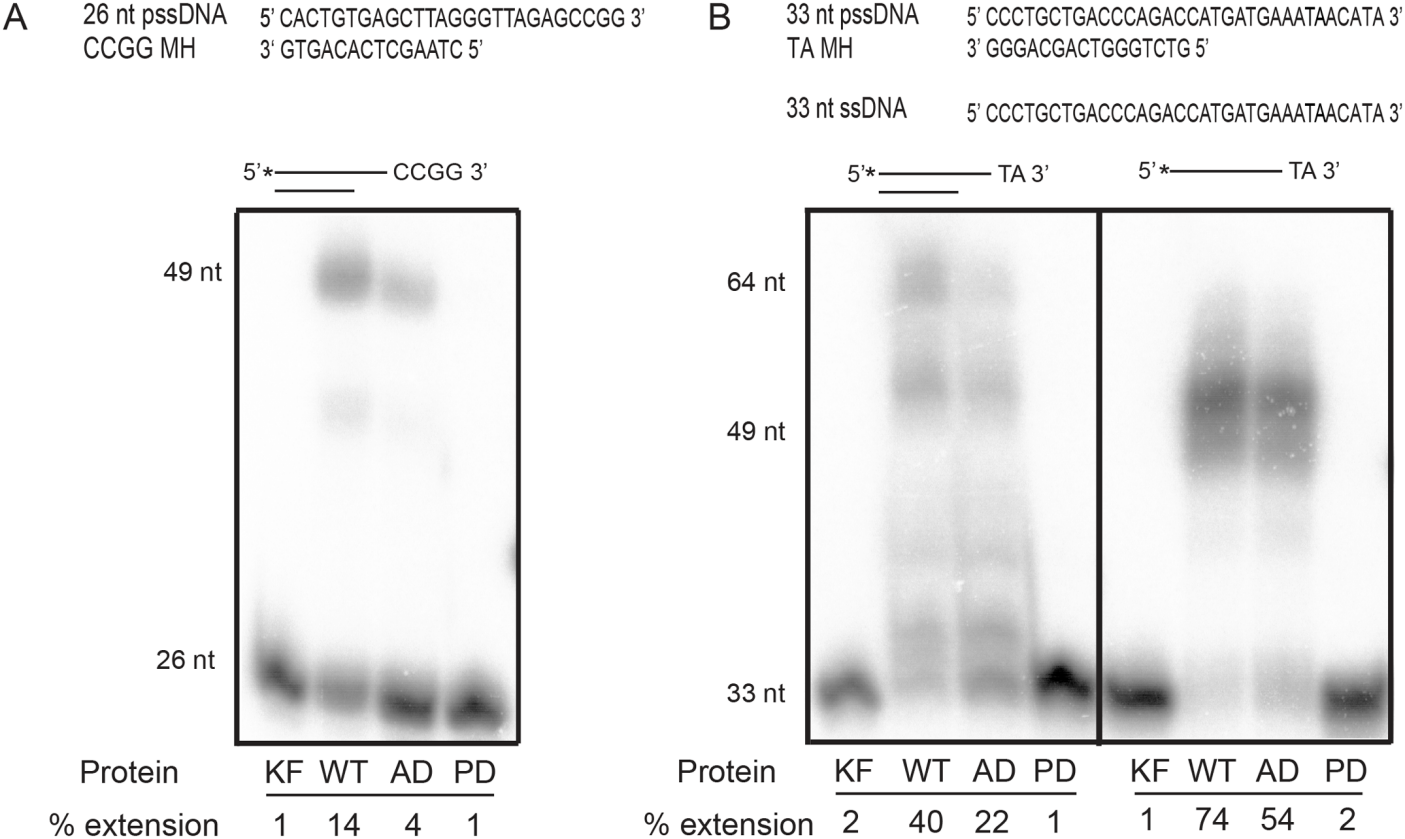
Pol θ ATPase activity is important for annealing and extension reactions *in vitro*. **A.** Pol θ promotes annealing of partial single-stranded DNA (pssDNA) at terminal microhomologies and DNA synthesis. 26 nt pssDNA with a CCGG terminal microhomology is from [12]. 30 nM of pssDNA was incubated with 50 pM of Klenow fragment (KF) or wild-type (WT), ATPase-dead (AD), or Pol-dead (PD) Pol θ protein for 30 min at 37°C. **B.** Pol θ can promote inter- and intra-molecular annealing and extension reactions on pss and ssDNA. 33 nt pssDNA with a TA terminal microhomology corresponds to the DNA product created by *P{w*^*a*^*}* excision. 33 nt ssDNA is the top strand of 33 nt pssDNA. 30 nM of pssDNA or ssDNA was incubated with 50 pM of protein for 30 min at 37°C. All products were separated by denaturing PAGE on a 20% gel. Percent extension was calculated by measuring band intensities of all primer extension products and dividing by total intensity of all bands in the lane.

To further test whether the ATPase activity of Pol θ might be important for the complex insertions observed during alternative end joining, we utilized purified proteins with substrates that can only support templated DNA synthesis following annealing of 2 nt or longer terminal microhomologies. We began with a partial single-stranded DNA substrate that was previously used with the human Pol θ polymerase domain to simulate MMEJ-like synthesis reactions [12] (Fig 4A). Similar to the human protein, wild-type Drosophila Pol θ can carry out DNA synthesis with this substrate (Fig 4A). The size of the product is consistent with annealing of two molecules of the substrate at CCGG microhomologies, followed by extension and strand displacement by Pol θ. Interestingly, the amount of product is reduced with the ATPase-dead Pol θ (Fig 4A).

Next, we tested the ability of Pol θ to utilize a partial single-stranded DNA substrate mimicking the intermediates formed in the *P{w*^*a*^*}* assay (Fig 4B). Surprisingly, wild-type Pol θ was able to catalyze more extension with this substrate, even though the terminal microhomology was only a 2-nt TA. While the products formed in this reaction were more variable in size, the length of the largest products was consistent with intermolecular annealing of two substrate molecules at the TA terminal microhomologies and strand-displacement synthesis to the end of the template. Repeated attempts to clone and sequence the reaction products were unsuccessful. Therefore, while the smaller products likely result from annealing at other microhomologous sequences in the substrate, it is unknown whether these are intermolecular or intramolecular events. Similar to the results with the first substrate, ATPase-dead Pol θ produced fewer extension products, particularly the full-length products (Fig 4B).

The polymerase domain of human Pol θ has more MMEJ activity on partial single-stranded substrates compared to single-stranded oligonucleotides with similar terminal microhomologies [12]. However, when we tested the ability of both full-length and ATPase-dead Drosophila Pol θ to utilize a single-stranded oligonucleotide corresponding to one strand of the *P{w*^*a*^*}* substrate, we observed an enhanced extension activity compared to that observed with the partial single-stranded substrate (Fig 4B). The sizes of the products with the single-stranded substrate were smaller than what would be expected with intermolecular annealing at the TA microhomology and synthesis to the end of the template. Taken together, these data suggest that Drosophila Pol θ can utilize short microhomologies present in partially and fully single-stranded DNA as primers for DNA synthesis and that ATPase activity from the helicase-like domain promotes the efficiency of these reactions.

## Discussion

Interest in Pol θ-mediated DNA repair has grown following observations that Pol θ is significantly overexpressed in a number of different cancer types and elevated Pol θ levels correspond to poorer patient outcomes [35–41]. Intriguingly, human Pol θ has been shown to compete with HR *in vivo* and overexpression of Pol θ in HR-proficient non-tumor cells causes increased DNA damage foci, suggesting that high levels of Pol θ may promote genome instability [14, 38]. The relationship between Pol θ and tumorigenesis makes Pol θ an appealing target for cancer therapeutics, especially because HR-deficient tumors are particularly dependent on Pol θ-mediated repair [14, 42]. Therefore, understanding the mechanisms by which Pol θ uses its dual-domain structure to repair DSBs and promote repair/bypass of other types of DNA damage is critical in order to identify possible Pol θ enzymatic targets. In this study, we have demonstrated important roles for both the ATPase and polymerase activities of Drosophila Pol θ during ICL bypass and alternative end-joining repair. Since the domains containing both of these enzymatic functions are physically connected and conserved in metazoans, this implies that the two domains may cooperate during these important processes.

### What is Pol θ doing to promote ICL tolerance?

One possible role for Pol θ in ICL tolerance is the bypass of unhooked crosslinked duplex DNA following single-stranded nicking by nucleases [30]. The polymerase active site of Pol θ contains several features that make it an ideal enzyme to bypass bulky lesions such as crosslinks, including unique insertion loops that provide a stable interaction between a poorly matched primer and template strand [43]. Here, we have shown that Drosophila Pol θ is able to bypass model unhooked ICL substrates *in vitro*. Specifically, it can insert a nucleotide opposite a crosslinked base and subsequently extend from the insertion point, in the context of duplexes of variable lengths (6–20 bp) duplexes surrounding the ICL. To date, only polymerase eta has been shown to carry out extension of these substrates with similar efficiency [30, 31].

Interestingly, while the ATPase activity of Drosophila Pol θ is required for ICL tolerance *in vivo*, it plays only a minor role during bypass *in vitro*. Perhaps ATP hydrolysis is only required for efficient bypass of an ICL in a cellular context, where the helicase-like domain could be required to displace other proteins from the site of the lesion. In support of this, protein displacement is a major function of several other SF1 and SF2 helicases [44–48].

Alternatively, Pol θ ATPase activity might be necessary for efficient repair of one or two-ended DSBs that form during intermediate stages of ICL repair [1]. Our mutagen sensitivity assays were performed by treating larvae with nitrogen mustard during a time in larval development when imaginal disc cells, which are necessary for metamorphosis and adult survival, are undergoing rapid divisions. Mutations that result in an inability to repair DSBs during this developmental window will cause organismal death. A recent study using zebrafish found that Pol θ-mediated end joining is most critical at the earliest stages of blastulation, when cells divide every 15 minutes [21]. Thus, Pol θ-mediated end joining may be particularly crucial for the repair of crosslink-related DSBs that occur in rapidly dividing cells [49].

Although Pol θ is essential for ICL tolerance in Drosophila, Arabidopsis, and *C*. *elegans*, it is not required in mouse or human cell lines [5, 11, 50, 51]. A possible explanation for this difference is that another TLS polymerase with redundant activity might substitute for Pol θ in mammals. One potential candidate is Pol ν, which has been suggested to participate in ICL repair [52]; human cell lines lacking Pol ν are sensitive to ICL-inducing agents [53, 54]. Intriguingly, the organisms which utilize Pol θ for ICL tolerance (Drosophila, Arabidopsis, and *C*. *elegans*) lack a Pol ν homolog [55]. However, recent reports also demonstrate that recombinant human Pol ν cannot bypass cisplatin-induced crosslinks *in vitro* [56], inconsistent with a substitution model.

### Roles of Pol θ during alternative end joining

We found that mutations that abolish the ATPase activity of Pol θ do not increase sensitivity to IR-induced DNA damage. Additionally, loss of ATPase activity does not change the frequency of Pol θ-mediated end-joining repair following creation of a site-specific break. This reflects a similarity between Drosophila and mammalian cells, which also require polymerase activity, but not the helicase-like domain, for Pol θ-mediated end joining [12, 13].

Pol θ has also been shown to generate templated insertions during alternative end joining [13, 17, 19, 57]. These insertions might result from a cell’s attempt to generate microhomologies suitable for alternative end joining with initially incompatible DNA ends. Strikingly, when analyzing our repair junction sequences, we found that the ATPase-dead mutants had a significant, 8-fold reduction in insertion events compared to the controls and a corresponding increase in the number of repair events using 2–3 nt microhomologies. Similarly, purified Pol θ lacking ATPase activity was less efficient at annealing and extending single-stranded DNA substrates.

Previous biochemical studies have established that the polymerase domain of human Pol θ aligns substrates with long GC-rich microhomologies more efficiently than AT-rich microhomologies and prefers using terminal microhomologies over internal ones [12]. We observed a similar predisposition with our partial single-stranded substrates. However, in the *in vivo P{w*^*a*^*}* repair system, end joining occurs using a 17 nucleotide AT-rich 3’ overhang. We see frequent usage of a long, 10-nt microhomology internal to the break site during junction formation. Utilization of internal microhomologies has also been observed in human cells [23]. Though our sequence is quite AT-rich, the most frequently used internal microhomologies have several GCs, supporting the idea that Pol θ more readily aligns GC-rich sequences. Additionally, in our system complex insertion events are typically templated from sequences directly adjacent to the break site. One potential explanation for this is that the AT-rich nature of the overhang results in less stable annealing between DNA ends, promoting multiple rounds of synthesis.

*In vitro*, the human Pol θ polymerase domain efficiently uses partially single-stranded DNA with a 3’ overhang as a substrate for MMEJ, while fully ssDNA is used much less efficiently [12]. While we do observe annealing and extension products with partially single-stranded DNA, we found that full length Drosophila Pol θ robustly uses ssDNA as a template for synthesis *in vitro*. Because we failed to observe products consistent with intermolecular annealing at a TA terminal microhomology (Fig 4C), we postulate that Pol θ may also use intramolecular hairpin-like structures as a template for synthesis, in a process that we have called synthesis-dependent microhomology-mediated end joining [25]. Similar observations have been made by others studying human Pol θ [57].

## Conclusion

While we have learned much about the roles of Pol θ in alternative end joining and ICL tolerance from biochemical studies, our findings illustrate that its functions *in vivo* are likely affected by additional factors. These might include variations in DNA sequence context or chromatin structure near DNA lesions and by interactions with other proteins that remain to be identified. Furthermore, the higher-order structure of Pol θ *in vivo* is likely important for its function. Crystal structures of the polymerase and helicase-like regions of Pol θ suggest that these subdomains can exist as dimers and tetramers, respectively [28, 43], but the oligomeric state of the full-length protein necessary for its *in vivo* functions is currently unknown.

In summary, our studies with full-length Drosophila Pol θ suggest that the helicase-like and polymerase domains of Pol θ play important roles during both ICL repair and alternative end joining. Given the frequent overexpression of Pol θ in human cancers, our findings highlight the potential utility of therapeutically targeting one or both domains to modulate its activity [14].

## Materials and methods

### Fly stocks and mutant creation

Fly stocks were maintained on standard cornmeal agar medium at 25°C. The *mus308*^*D2*^, *spn-A*^*057*^, and *spn-A*^*093*^ alleles were obtained from Bloomington Stock Center. The *mus308Δ* allele was generated through an imprecise excision screen of the *P*-element *P{GSV7}GS23034* (Kyoto Drosophila Genomics and Genetics Resources stock center). The *mus308Δ* allele deletes 14,250 bp spanning *mus308*, its endogenous promoter, and part of the neighboring gene *Men*. To generate the transgenic alleles, genomic DNA encoding *mus308* and 1.25 kb of upstream sequence, including its endogenous promoter, was isolated and amplified with primers containing BglII and Acc65I restriction enzyme sites. The DNA was cloned into the pMTL vector and mutagenized through amplification-based site-specific mutagenesis. Mutagenized alleles were then cloned into the pattB expression vector. The plasmids were injected into embryos containing attP integration sites by BestGene injection services and genome integration was achieved through the Fly-C31 lambda phage recombination system [58, 59]. Transformants were identified using a *white*^+^ marker present on pattB. Control, polymerase-dead, and ATPase-dead transgenes were inserted at the *ZH-attp-51C* site (Bloomington stock number 24483) on chromosome *2*.

### Mutagen sensitivity assays

For nitrogen mustard assays, 5–8 *mus308 transgene*; *mus308Δ/TM6B* female flies were mated with 3–4 *mus308 transgene*; *mus308Δ/TM6B* males flies in a standard vial containing 5 mL cornmeal agar. Flies were allowed to mate and lay eggs for 3 days before being transferred to fresh vials for an additional 2 days. The first set of vials was treated with 250 μL of nitrogen mustard solution while the second control set was treated with 250 μL water. For irradiation assays, 40–50 *mus308 transgene; mus308Δ*, *spnA*^*057*^*/TM3* females were mated to 10–15 *mus308Δ*, *spnA*^*093*^*/TM6B* males and eggs were collected on grape juice agar plates for 12 hours. Eggs were allowed to hatch and mature to third instar larvae, then irradiated using a Gammator 1000 irradiator. Percent relative survival was calculated using the ratio of *mus308* homozygous to heterozygous adult survival in mutagen treated vials compared to control vials. Each experiment consisted of at least 5 independent vials or 1 grape agar plate and experiments were repeated in triplicate.

### *P{w*^*a*^*}* gap repair assay

End joining repair of a double strand break was monitored after the excision of a *P{w*^*a*^*}* element as described previously [60]. A second chromosome transposase source (*CyO*, *H{w+*, *Δ2–3}*) was used to excise *P{w*^*a*^*}*. Single males of the genotype *P{w*^*a*^*}; mus308 transgene/CyO*, *H{w+*, *Δ2–3}; mus308Δ*, *spnA*^*057*^*/mus308*^*D2*^, *spnA*^*093*^ were mated to *P{w*^*a*^*}* females and individual repair events were recovered in the female progeny. Progeny containing end-joining events lose a functional copy of the *white* gene, thus end-joining repair events can be quantified and recovered in female progeny with yellow eyes. For each genotype, individual male crosses were scored for eye color of female progeny. The percentage of progeny from each repair class was calculated on a per vial basis, with each vial representing an independent experiment. Statistical comparisons were done with a non-parametric ANOVA followed by Tukey’s test using InStat3 (GraphPad).

### Repair junction sequencing

Female progeny containing the repaired *P{w*^*a*^*}* construct were collected from the single male crosses. To isolate genomic DNA, whole flies were manually disrupted in 50 uL squishing buffer (10 mM Tris-HCl pH8, 1 mM EDTA, 25 mM NaCl, 200 g/ml Proteinase K) and incubated at 37°C for 30 minutes then 95°C for 3 minutes. Repair junctions were amplified by PCR using primers near the junction Sd5320 (ACCATTGCAAGCTACATAGCTGAC) and Sd5941R (GCCTTGCTTCTTCCACACAGCGTG). PCR products were sequenced using the Sd5320 primer.

### Pol θ protein purification

Full-length *mus308* cDNA (Drosophila Genomics Resource Center, clone LP14642) was amplified with primers containing SalI and Acc65I restriction sites and cloned into pFastbac1 (Invitrogen, gift from Timur Yusufzai) in frame with the 6X His and FLAG tags to make pFBFL308. ATPase-dead and pol-dead mutant clones were created by site-directed mutagenesis using Q5 polymerase (NEB, Ipswich, MA). These constructs were used with the Bac to Bac Baculovirus Expression System (Life Technologies) for expression in Sf9 cells (Orbigen, San Diego, CA.)

Sf9 cells (1 L, 6.0 × 10^6^ cells/ml) were infected with each construct for 72 hr at 25°C and harvested by centrifugation. Cells were sonicated in lysis buffer containing 20 mM Hepes, pH 7.6, 500 mM NaCl, 1.5 mM MgCl2, 10% glycerol, 0.1% Triton X-100, 1 mM phenylmethylsulfonyl fluoride (PMSF), and complete EDTA-free protease inhibitor (Roche), with three 10 second bursts at 20% duty cycle with 10 seconds rest between bursts. Debris was removed by centrifugation and the supernatant was incubated for 4 hr at 4°C with 200 μl of anti-DYKDDDDK G1 affinity resin (Genscript). The resin was washed with 10 volumes of the lysis buffer and eluted with 250 μg/ml 3X FLAG peptide (gift of S. Fuchs). The eluate was spun through a Zeba 40K desalting column (ThermoFisher) following the manufacturer’s directions. Protein amounts were quantified by comparison with a BSA standard curve following transfer from an SDS-PAGE gel using Fastblot stain (G Biosciences).

### ICL bypass assays

5-atom ICL-containing substrates were synthesized according to (Roy et al., 2016[61]). These were annealed with P15 (5’-CACTGACTCTATGATG-3’) labeled at the 5’ end with 6-FAM. ICL substrates (150 nM) and 5’ labeled primer P15 (50 nM) were annealed in 10 mM Tris-HCl pH 8.0, 50 mM NaCl, overnight at room temperature to ensure the stability of the ICLs. The ICL substrates/primers (5 nM, with respect to the primer) were incubated with Klenow (exo-) fragment or Pol θ in a reaction volume of 10 μL. For assays with Klenow (exo-), 1 nM enzyme was used in reaction buffer NEB2 (50 mM NaCl, 10 mM Tris-HCl, 10 mM MgCl_2_, 1 mM DTT). 0.2 nM Pol θ was used in a reaction buffer containing 25 mM Tris-Cl pH 7.5, 10 mM MgCl_2_, 200 μM ATP, 100 μg/mL BSA and 4.8% glycerol. Reactions with Klenow (exo-) were incubated for 5 minutes, and with Pol θ for 10 minutes at 37°C. Reactions were stopped by addition of 10 μL of formamide buffer (80% formamide, 1 mM EDTA, 1 mg/mL Orange G), denatured at 95°C for 2 minutes and chilled on ice. The products of the reaction were resolved on a 10% 7M Urea PAGE and FAM labeled DNA was visualized using a Typhoon 9400 scanner (GE Healthcare). Images were analyzed and quantified using ImageQuant software (Molecular Dynamics)

### Pol θ polymerase assays

The polymerase assay was adapted from [8]. To create the 26nt pssDNA substrate with CCGG microhomology, PAGE-purified oligo 5’-CTAAGCTCACAGTG-3’ (IDT) was 5’ end-labeled with polynucleotide kinase (NEB) and ATP, [γ-32P]- 3000Ci/mmol 10mCi/ml (Perkin-Elmer). The labeled oligo was annealed to an oligo of sequence 5’-CACTGTGAGCTTAGGGTTAGAGCCGG-3’ in STE buffer (100 mM NaCl, 10mM Tris-HCl, pH 8.0, 1 mM EDTA) by heating to 85°C and slowly cooling to room temperature. To create the 33nt pssDNA substrate with TA microhomology, PAGE-purified oligos 5’-GTCTGGGTCAGCAGGG-3’ and 5’-CCCTGCTGACCCAGACCATGATGAAATAACATA-3’ were annealed under identical conditions. Reaction mixtures (20 μl) contained 20 mM Tris–HCl pH 7.5, 4% glycerol, 80 μg/ml bovine serum albumin (BSA), 8 mM MgCl_2_, 16 fmol of substrate, 100 μM dNTPs, and 0.25–0.5 ng of Pol θ. After incubation for 10 min at 37°C, reactions were terminated by adding gel loading buffer (formamide, 0.1% xylene cyanol, 0.1% bromophenol blue, 20 mM EDTA) and boiling. Products were separated by 20% denaturing SDS-PAGE and band intensities were analyzed with a Biorad phosphorimager.

## Supporting information

S1 Fig Purification of FLAG-tagged Drosophila Pol θ. **(A) FLAG-tagged wild-type (WT), ATPase-dead (AD), and polymerase-dead (PD) Pol θ was affinity purified using FLAG resin and the eluate was spun through a 40K desalting column**. Shown is a nitrocellulose membrane stained with Fastblot following SDS-PAGE and transfer. (B) Western blot of the membrane probed with anti-FLAG antibody.
(PDF)

S1 Table Loss of polymerase, but not ATPase activity, results in deletion-prone alt-EJ repair. The *P{w*^*a*^*}* element is inserted into an intron of the *scalloped* (*Sd*) gene. Following *P{w*^*a*^*}* excision, repair events from the male germline that delete more than 1.5 kb of flanking sequence result in *sd-* alleles that cause a scalloped-wing phenotype in female progeny.
(PDF)

## References

[1] Muniandy PA, Liu J, Majumdar A, Liu ST, Seidman MM. DNA interstrand crosslink repair in mammalian cells: step by step. Crit Rev Biochem Mol Biol. 2010;45(1):23–49. 10.3109/10409230903501819 ; 20039786 PMC2824768

[2] Deans AJ, West SC. DNA interstrand crosslink repair and cancer. Nat Rev Cancer. 2011;11(7):467–80. 10.1038/nrc3088 ; 21701511 PMC3560328

[3] Haynes B, Saadat N, Myung B, Shekhar MP. Crosstalk between translesion synthesis, Fanconi anemia network, and homologous recombination repair pathways in interstrand DNA crosslink repair and development of chemoresistance. Mutat Res Rev Mutat Res. 2015;763:258–66. 10.1016/j.mrrev.2014.11.005 ; 25795124 PMC4369322

[4] Howard SM, Yanez DA, Stark JM. DNA damage response factors from diverse pathways, including DNA crosslink repair, mediate alternative end joining. PLoS Genet. 2015;11(1):e1004943 10.1371/journal.pgen.1004943 ; 25629353 PMC4309583

[5] Boyd JB, Sakaguchi K, Harris PV. mus308 mutants of Drosophila exhibit hypersensitivity to DNA cross-linking agents and are defective in a deoxyribonuclease. Genetics. 1990;125(4):813–9. ; 2397884 10.1093/genetics/125.4.813 PMC1204107

[6] Leonhardt EA, Henderson DS, Rinehart JE, Boyd JB. Characterization of the mus308 gene in Drosophila melanogaster. Genetics. 1993;133(1):87–96. 8417992 10.1093/genetics/133.1.87 PMC1205301

[7] Harris PV, Mazina OM, Leonhardt EA, Case RB, Boyd JB, Burtis KC. Molecular cloning of Drosophila mus308, a gene involved in DNA cross-link repair with homology to prokaryotic DNA polymerase I genes. Molecular and cellular biology. 1996;16(10):5764–71. 8816490 10.1128/mcb.16.10.5764 PMC231577

[8] Seki M, Marini F, Wood RD. POLQ (Pol theta), a DNA polymerase and DNA-dependent ATPase in human cells. Nucleic Acids Res. 2003;31(21):6117–26. ; 10.1093/nar/gkg814 14576298 PMC275456

[9] Sharief FS, Vojta PJ, Ropp PA, Copeland WC. Cloning and chromosomal mapping of the human DNA polymerase theta (POLQ), the eighth human DNA polymerase. Genomics. 1999;59(1):90–6. 10.1006/geno.1999.5843 . 10395804

[10] Yousefzadeh MJ, Wood RD. DNA polymerase POLQ and cellular defense against DNA damage. DNA repair. 2013;12(1):1–9. 10.1016/j.dnarep.2012.10.004 23219161 PMC3534860

[11] Muzzini DM, Plevani P, Boulton SJ, Cassata G, Marini F. Caenorhabditis elegans POLQ-1 and HEL-308 function in two distinct DNA interstrand cross-link repair pathways. DNA repair. 2008;7(6):941–50. 10.1016/j.dnarep.2008.03.021 18472307

[12] Kent T, Chandramouly G, McDevitt SM, Ozdemir AY, Pomerantz RT. Mechanism of microhomology-mediated end-joining promoted by human DNA polymerase θ. Nat Struct Mol Biol. 2015;22(3):230–7. 10.1038/nsmb.2961 ; 25643323 PMC4351179

[13] Yousefzadeh MJ, Wyatt DW, Takata K, Mu Y, Hensley SC, Tomida J, et al Mechanism of suppression of chromosomal instability by DNA polymerase POLQ. PLoS Genet. 2014;10(10):e1004654 10.1371/journal.pgen.1004654 ; 25275444 PMC4183433

[14] Ceccaldi R, Liu JC, Amunugama R, Hajdu I, Primack B, Petalcorin MI, et al Homologous-recombination-deficient tumours are dependent on Polθ-mediated repair. Nature. 2015;518(7538):258–62. 10.1038/nature14184 . 25642963 PMC4415602

[15] Beagan K, McVey M. Linking DNA polymerase theta structure and function in health and disease. Cell Mol Life Sci. 2016;73(3):603–15. 10.1007/s00018-015-2078-9 ; 26514729 PMC4715478

[16] Black SJ, Kashkina E, Kent T, Pomerantz RT. DNA Polymerase theta: A Unique Multifunctional End-Joining Machine. Genes (Basel). 2016;7(9). 10.3390/genes7090067 ; 27657134 PMC5042397

[17] Mateos-Gomez PA, Gong F, Nair N, Miller KM, Lazzerini-Denchi E, Sfeir A. Mammalian polymerase θ promotes alternative NHEJ and suppresses recombination. Nature. 2015;518(7538):254–7. 10.1038/nature14157 . 25642960 PMC4718306

[18] Chan SH, Yu AM, McVey M. Dual roles for DNA polymerase theta in alternative end-joining repair of double-strand breaks in Drosophila. PLoS genetics. 2010;6(7):e1001005 10.1371/journal.pgen.1001005 20617203 PMC2895639

[19] Koole W, van Schendel R, Karambelas AE, van Heteren JT, Okihara KL, Tijsterman M. A Polymerase Theta-dependent repair pathway suppresses extensive genomic instability at endogenous G4 DNA sites. Nat Commun. 2014;5:3216 10.1038/ncomms4216 . 24496117

[20] Roerink SF, van Schendel R, Tijsterman M. Polymerase theta-mediated end joining of replication-associated DNA breaks in C. elegans. Genome Res. 2014;24(6):954–62. 10.1101/gr.170431.113 ; 24614976 PMC4032859

[21] Thyme SB, Schier AF. Polq-Mediated End Joining Is Essential for Surviving DNA Double-Strand Breaks during Early Zebrafish Development. Cell Rep. 2016 10.1016/j.celrep.2016.03.072 . 27149851 PMC5063659

[22] Truong LN, Li Y, Shi LZ, Hwang PY, He J, Wang H, et al Microhomology-mediated End Joining and Homologous Recombination share the initial end resection step to repair DNA double-strand breaks in mammalian cells. Proc Natl Acad Sci U S A. 2013;110(19):7720–5. 10.1073/pnas.1213431110 ; 23610439 PMC3651503

[23] Wyatt DW, Feng W, Conlin MP, Yousefzadeh MJ, Roberts SA, Mieczkowski P, et al Essential Roles for Polymerase theta-Mediated End Joining in the Repair of Chromosome Breaks. Mol Cell. 2016;63(4):662–73. 10.1016/j.molcel.2016.06.020 ; 27453047 PMC4992412

[24] Sfeir A, Symington LS. Microhomology-Mediated End Joining: A Back-up Survival Mechanism or Dedicated Pathway? Trends Biochem Sci. 2015;40(11):701–14. 10.1016/j.tibs.2015.08.006 ; 26439531 PMC4638128

[25] Yu AM, McVey M. Synthesis-dependent microhomology-mediated end joining accounts for multiple types of repair junctions. Nucleic acids research. 2010;38(17):5706–17. 10.1093/nar/gkq379 20460465 PMC2943611

[26] van Schendel R, van Heteren J, Welten R, Tijsterman M. Genomic Scars Generated by Polymerase Theta Reveal the Versatile Mechanism of Alternative End-Joining. PLoS Genet. 2016;12(10):e1006368 10.1371/journal.pgen.1006368 ; 27755535 PMC5068794

[27] van Kregten M, de Pater S, Romeijn R, van Schendel R, Hooykaas PJ, Tijsterman M. T-DNA integration in plants results from polymerase-theta-mediated DNA repair. Nat Plants. 2016;2(11):16164 10.1038/nplants.2016.164 . 27797358

[28] Newman JA, Cooper CD, Aitkenhead H, Gileadi O. Structure of the Helicase Domain of DNA Polymerase Theta Reveals a Possible Role in the Microhomology-Mediated End-Joining Pathway. Structure. 2015;23(12):2319–30. 10.1016/j.str.2015.10.014 ; 26636256 PMC4671958

[29] Zhang J, Walter JC. Mechanism and regulation of incisions during DNA interstrand cross-link repair. DNA Repair (Amst). 2014;19:135–42. 10.1016/j.dnarep.2014.03.018 ; 24768452 PMC4076290

[30] Roy U, Scharer OD. Involvement of translesion synthesis DNA polymerases in DNA interstrand crosslink repair. DNA Repair (Amst). 2016;44:33–41. 10.1016/j.dnarep.2016.05.004 . 27311543 PMC5524570

[31] Roy U, Mukherjee S, Sharma A, Frank EG, Scharer OD. The structure and duplex context of DNA interstrand crosslinks affects the activity of DNA polymerase eta. Nucleic Acids Res. 2016;44(15):7281–91. 10.1093/nar/gkw485 ; 27257072 PMC5009737

[32] Ho TV, Guainazzi A, Derkunt SB, Enoiu M, Scharer OD. Structure-dependent bypass of DNA interstrand crosslinks by translesion synthesis polymerases. Nucleic Acids Res. 2011;39(17):7455–64. 10.1093/nar/gkr448 ; 21666254 PMC3177197

[33] McVey M. In vivo analysis of Drosophila BLM helicase function during DNA double-strand gap repair. Methods Mol Biol. 2010;587:185–94. 10.1007/978-1-60327-355-8_13 . 20225150

[34] McVey M, Larocque JR, Adams MD, Sekelsky JJ. Formation of deletions during double-strand break repair in Drosophila DmBlm mutants occurs after strand invasion. Proc Natl Acad Sci U S A. 2004;101(44):15694–9. 10.1073/pnas.0406157101 ; 15501916 PMC524851

[35] Allera-Moreau C, Rouquette I, Lepage B, Oumouhou N, Walschaerts M, Leconte E, et al DNA replication stress response involving PLK1, CDC6, POLQ, RAD51 and CLASPIN upregulation prognoses the outcome of early/mid-stage non-small cell lung cancer patients. Oncogenesis. 2012;1:e30 10.1038/oncsis.2012.29 ; 23552402 PMC3503291

[36] Higgins GS, Harris AL, Prevo R, Helleday T, McKenna WG, Buffa FM. Overexpression of POLQ confers a poor prognosis in early breast cancer patients. Oncotarget. 2010;1(3):175–84. 10.18632/oncotarget.124 20700469 PMC2917771

[37] Kawamura K, Bahar R, Seimiya M, Chiyo M, Wada A, Okada S, et al DNA polymerase theta is preferentially expressed in lymphoid tissues and upregulated in human cancers. Int J Cancer. 2004;109(1):9–16. 10.1002/ijc.11666 . 14735462

[38] Lemee F, Bergoglio V, Fernandez-Vidal A, Machado-Silva A, Pillaire MJ, Bieth A, et al DNA polymerase theta up-regulation is associated with poor survival in breast cancer, perturbs DNA replication, and promotes genetic instability. Proceedings of the National Academy of Sciences of the United States of America. 2010;107(30):13390–5. 10.1073/pnas.0910759107 20624954 PMC2922118

[39] Lessa RC, Campos AH, Freitas CE, Silva FR, Kowalski LP, Carvalho AL, et al Identification of upregulated genes in oral squamous cell carcinomas. Head Neck. 2013;35(10):1475–81. 10.1002/hed.23169 . 22987617

[40] Li WQ, Hu N, Hyland PL, Gao Y, Wang ZM, Yu K, et al Genetic variants in DNA repair pathway genes and risk of esophageal squamous cell carcinoma and gastric adenocarcinoma in a Chinese population. Carcinogenesis. 2013;34(7):1536–42. 10.1093/carcin/bgt094 ; 23504502 PMC3697889

[41] Pillaire MJ, Selves J, Gordien K, Gourraud PA, Gouraud PA, Gentil C, et al A 'DNA replication' signature of progression and negative outcome in colorectal cancer. Oncogene. 2010;29(6):876–87. 10.1038/onc.2009.378 . 19901968

[42] Wood RD, Doublie S. DNA polymerase theta (POLQ), double-strand break repair, and cancer. DNA Repair (Amst). 2016;44:22–32. 10.1016/j.dnarep.2016.05.003 ; 27264557 PMC5114520

[43] Zahn KE, Averill AM, Aller P, Wood RD, Doublié S. Human DNA polymerase θ grasps the primer terminus to mediate DNA repair. Nat Struct Mol Biol. 2015 10.1038/nsmb.2993 . 25775267 PMC4385486

[44] Byrd AK, Raney KD. Protein displacement by an assembly of helicase molecules aligned along single-stranded DNA. Nat Struct Mol Biol. 2004;11(6):531–8. 10.1038/nsmb774 . 15146172

[45] Byrd AK, Raney KD. Displacement of a DNA binding protein by Dda helicase. Nucleic Acids Res. 2006;34(10):3020–9. 10.1093/nar/gkl369 ; 16738140 PMC1474059

[46] Florés MJ, Sanchez N, Michel B. A fork-clearing role for UvrD. Mol Microbiol. 2005;57(6):1664–75. 10.1111/j.1365-2958.2005.04753.x . 16135232

[47] Macris MA, Sung P. Multifaceted role of the Saccharomyces cerevisiae Srs2 helicase in homologous recombination regulation. Biochem Soc Trans. 2005;33(Pt 6):1447–50. 10.1042/BST20051447 . 16246143

[48] Morris PD, Raney KD. DNA helicases displace streptavidin from biotin-labeled oligonucleotides. Biochemistry. 1999;38(16):5164–71. 10.1021/bi9822269 . 10213622

[49] Alexander JL, Beagan K, Orr-Weaver TL, McVey M. Multiple mechanisms contribute to double-strand break repair at rereplication forks in Drosophila follicle cells. Proc Natl Acad Sci U S A. 2016;113(48):13809–14. 10.1073/pnas.1617110113 ; 27849606 PMC5137741

[50] Inagaki S, Suzuki T, Ohto MA, Urawa H, Horiuchi T, Nakamura K, et al Arabidopsis TEBICHI, with helicase and DNA polymerase domains, is required for regulated cell division and differentiation in meristems. Plant Cell. 2006;18(4):879–92. 10.1105/tpc.105.036798 ; 16517762 PMC1425847

[51] Shima N, Munroe RJ, Schimenti JC. The mouse genomic instability mutation chaos1 is an allele of Polq that exhibits genetic interaction with Atm. Mol Cell Biol. 2004;24(23):10381–9. 10.1128/MCB.24.23.10381-10389.2004 ; 15542845 PMC529050

[52] Ho TV, Scharer OD. Translesion DNA synthesis polymerases in DNA interstrand crosslink repair. Environ Mol Mutagen. 2010;51(6):552–66. 10.1002/em.20573 . 20658647

[53] Moldovan GL, Madhavan MV, Mirchandani KD, McCaffrey RM, Vinciguerra P, D'Andrea AD. DNA polymerase POLN participates in cross-link repair and homologous recombination. Mol Cell Biol. 2010;30(4):1088–96. 10.1128/MCB.01124-09 ; 19995904 PMC2815579

[54] Yamanaka K, Minko IG, Takata K, Kolbanovskiy A, Kozekov ID, Wood RD, et al Novel enzymatic function of DNA polymerase nu in translesion DNA synthesis past major groove DNA-peptide and DNA-DNA cross-links. Chem Res Toxicol. 2010;23(3):689–95. 10.1021/tx900449u ; 20102227 PMC2838406

[55] Marini F, Kim N, Schuffert A, Wood RD. POLN, a nuclear PolA family DNA polymerase homologous to the DNA cross-link sensitivity protein Mus308. J Biol Chem. 2003;278(34):32014–9. 10.1074/jbc.M305646200 . 12794064

[56] Takata K, Shimizu T, Iwai S, Wood RD. Human DNA polymerase N (POLN) is a low fidelity enzyme capable of error-free bypass of 5S-thymine glycol. J Biol Chem. 2006;281(33):23445–55. 10.1074/jbc.M604317200 . 16787914

[57] Kent T, Mateos-Gomez PA, Sfeir A, Pomerantz RT. Polymerase theta is a robust terminal transferase that oscillates between three different mechanisms during end-joining. Elife. 2016;5 10.7554/eLife.13740 ; 27311885 PMC4912351

[58] Huang J, Zhou W, Dong W, Hong Y. Targeted engineering of the Drosophila genome. Fly (Austin). 2009;3(4):274–7. . 19823033 10.4161/fly.9978

[59] Bischof J, Maeda RK, Hediger M, Karch F, Basler K. An optimized transgenesis system for Drosophila using germ-line-specific phiC31 integrases. Proc Natl Acad Sci U S A. 2007;104(9):3312–7. 10.1073/pnas.0611511104 ; 17360644 PMC1805588

[60] McVey M, Adams M, Staeva-Vieira E, Sekelsky JJ. Evidence for multiple cycles of strand invasion during repair of double-strand gaps in Drosophila. Genetics. 2004;167(2):699–705. 10.1534/genetics.103.025411 ; 15238522 PMC1470890

[61] Guainazzi A, Campbell AJ, Angelov T, Simmerling C, Scharer OD. Synthesis and molecular modeling of a nitrogen mustard DNA interstrand crosslink. Chemistry. 2010;16(40):12100–3. 10.1002/chem.201002041 20842675 PMC4870935

