# Supplementary material for: Drosophila DNA polymerase theta utilizes both helicase-like and polymerase domains during microhomology-mediated end joining and interstrand crosslink repair
Source: PLoS Genet. 2017 May 25;13(5):e1006813. doi: 10.1371/journal.pgen.1006813 (PMC5466332; doi:10.1371/journal.pgen.1006813)
Supplement: S1 Fig — (A) FLAG-tagged wild-type (WT), ATPase-dead (AD), and polymerase-dead (PD) Pol θ was affinity purified using FLAG resin and the eluate was spun through a 40K desalting column. Shown is a nitrocellulose membrane stained with Fastblot following SDS-PAGE and transfer. (B) Western blot of the membrane probed with anti-FLAG antibody. (PDF) [file pgen.1006813.s001.pdf]

S1 Fig

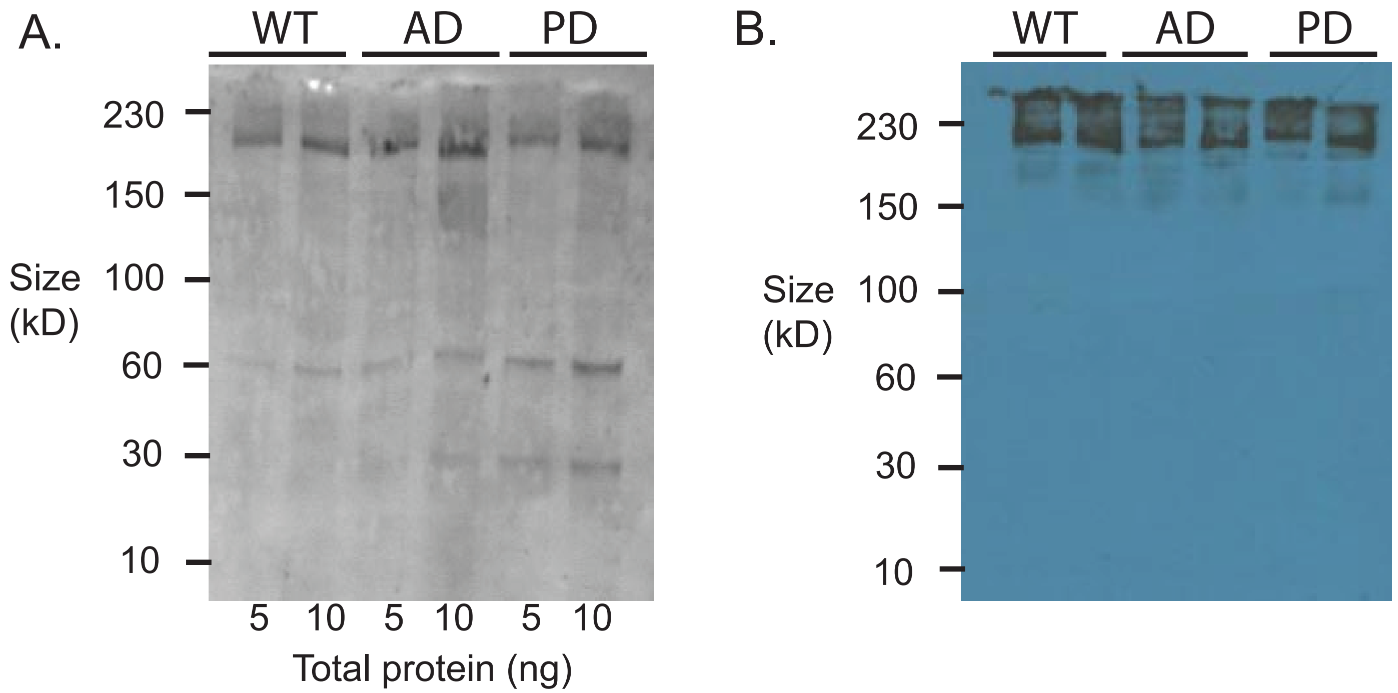

Figure S1. Purification of FLAG-tagged Drosophila Pol θ. (A) FLAG-tagged wild-type (WT), ATPase-dead (AD), and polymerase-dead (PD) Pol θ was affinity purified using FLAG resin and the eluate was spun through a 40K desalting column. Shown is a nitrocellulose membrane stained with Fastblot following SDS-PAGE and transfer. (B) Western blot of the membrane probed with anti-FLAG antibody.
