# Supplementary material for: Drosophila DNA polymerase theta utilizes both helicase-like and polymerase domains during microhomology-mediated end joining and interstrand crosslink repair
Source: PLoS Genet. 2017 May 25;13(5):e1006813. doi: 10.1371/journal.pgen.1006813 (PMC5466332; doi:10.1371/journal.pgen.1006813)
Supplement: S1 Table — The P{wa} element is inserted into an intron of the scalloped (Sd) gene. Following P{wa} excision, repair events from the male germline that delete more than 1.5 kb of flanking sequence result in sd- alleles that cause a scalloped-wing phenotype in female progeny. (PDF) [file pgen.1006813.s002.pdf]

**Genotype**	**Deletions into *Sd***	**Percent deletions**
**Full-length Pol θ**	0/67	0%
**Wildtype Pol θ**	0/31	0%
**ATPase-dead**	3/67	4.4%
**Pol-dead**	4/10	40%
**Null/ no transgene**	2/7	28.6%

Supplemental Table 1 – Loss of polymerase, but not ATPase activity, results in deletion-prone alt-EJ repair. The *P{w^{a}}* element is inserted into an intron of the *scalloped* (*Sd*) gene. Following *P{w^{a}}* excision, repair events from the male germline that delete more than 1.5 kb of flanking sequence result in *sd-* alleles that cause a scalloped-wing phenotype in female progeny.
